# Hospital Hygiene
*Additional Suggestions, with a view to the Improvement of Hospitals. By John Roberton. Manchester: 1858.Report of the Commissioners appointed to Inquire into the Regulations affecting the Sanitary Condition of the Army, and the Organisation of Military Hospitals. London: 1858.Beckman's History of Inventions, Discoveries, and Origins. By Johnson. London: H. G. Bohn. 1846.Netley Hospital: a Return to an Address of the House of Commons. 1857.An Account of the Diseases most frequent in the British Military Hospitals in Germany, from January 1761 to March 1763. By Donald Monro, M.D. London: 1764.The Census of Ireland for 1851: Report on the Status of Disease. 1854.


**Published:** 1858-07

**Authors:** 


### SANITARY REVIEW.

JULY 1858.

### HOSPITAL HYGIENE.*

THERE are few subjects, in the domain of history, more curious or interesting than those which relate to the origin and progress of hospitals for the reception and treatment of the sick poor. This history involves many points relating to the modern hospital,—to its construction, its objects, and its management. We might grow very learned, or, at all events, very lengthy (and length sometimes passes for learning), on this topic; but we are content to take up Beckman, whom we consider to have pithed this matter of history most thoroughly, and following him mainly, to state so much as shall bear on the prominent fact to be insisted on in this short essay,—viz.: that the modern hospital partakes largely of the defects of the hospital of the past, and that such defects are often directly mental and even opposed to the objects of the sick hospital of the present day.

The buildings called hospitals seem first to have been intended for the reception and convenience not of the sick, but of the healthy, and for a religious rather than for a temporal purpose. In so far as the learned Beckman is to be trusted, the idea is sustainable, that the primitive hospitals were erected by the clergy at holy places, or at stations on the way to them; whither pilgrims, coming from afar, might find

* Additional Suggestions, with a view to the Improvement of Hospitals. By JOHN ROBERTON. Manchester: 1858.

Report of the Commissioners appointed to Inquire into the Regulations affecting the Sanitary Condition of the Army, and the Organisation of Military Hospitals. London: 1858.

Beckman's History of Inventions, Discoveries, and Origins. By JOHNSON. London: H. G. Bohn. 1846.

Netley Hospital: a Return to an Address of the House of Commons. 1857.

An Account of the Diseases most frequent in the British Military Hospitals in Germany, from January 1761 to March 1763. By DONALD MONRO, M.D. London: 1764.

The Census of Ireland for 1851: Report on the Status of Disease. 1854.

tenancy and comfort in their self-sacrificial journey. In this way Palestine became the site of the earlier of these institutions. The pilgrimage to this—the goal of the pilgrim's expectations, was one of physical danger. In its boundaries no friendly Christian village nor Christian inn greeted the wayfarer; who, too often, therefore, worn out with his self-imposed obligations sank by the way, a martyr to his cherished superstition. To prevent these evils, as we say, the hands of the clergy erected pilgrim hospitals, where the wearied recruited their strength, and the faithful their hope. Jerome, perchance, set one of the first examples, by building an hospital at Bethlehem; while Paula his friend, anxious to follow in the labour, besprinkled the paths to that famous village with similar resting-places for the "devout idlers" who turned Bethlehemward. The design thus established admitted of easy imitation and extension. The protection of the hospital ought, surely, not to be confined to the pilgrim; the mere traveller would find equal advantage in the provision. It proved so; long previous to the eleventh century, so far had this view been realized, that hospitals—superintended by special brotherhoods, and often very liberally endowed by the princely of the earth—became common over the whole of Europe; affording aid and shelter alike to the barefooted enthusiast yearning for the sight of the manger or the sepulchre, and to the well-clothed merchant hnngry only for the dross of the silver and the gold.

The first *sick* hospitals were the offshoots of the hospitals of rest above described. They who, visiting the hospitals of rest, brought with them cutaneous maladies and pests, must needs be removed from the healthy and must have a dwelling for themselves—a pesthouse. The pesthouse, in its turn, admitted of imitation and extension. If a pesthouse were a convenience to a hospital or temporary home for pilgrims or travellers, how much more a convenience should it be to a town or a city! The idea took root, grew, and flourished; and the hospital for the sick became the fact of time.

One of the first sick hospitals, perhaps *the* first, was of English production. In 1070, Lanfranc, archbishop of Canterbury, built a hospital for the special purpose of receiving the sick, and so arranged it that one half of the institution was applied to the reception of men, the other of women. The example again became general, and sick hospitals began to appear wherever the monastery or cathedral marked the pretensions of the all powerful church. The sick hospitals were first placed under the management of the bishops, who ultimately gave them in charge to their deacons. Afterwards, when the hospitals of the Church were imitated by the lay populations, the bishops, by virtue of their office, claimed the right to thrust the episcopal nose into the layman's brick and mortar. As the power of the bishop waned, the nose was pulled forcibly back, and the admission claimed as a right became possible only by courtesy.

Thus far we have followed simply the history of Beckman, but it is clear that the learned historian was not conversant with Hibernian history. The commissioners who report on the status of diseases in Ireland, relate that long prior to the erections of the hospice of the ecclesiastic, there existed homes for the sick and wounded of the Emerald Isle; for when the regal residences of Tara and Emania existed, there was attached to the latter the "Teagh na Craibhe Ruadhe", or "House of the Crimson Branch", where the warriors of old hung up their arms and trophies; and near to this stood the "Broin Bearg", or "House of Sorrow", where the sick and wounded were provided for. Thus, before the introduction of Christianity into this island, we read of a building being set apart for the maintenance and recovery of the wounded and diseased.

In later times, when an epidemic cutaneous disease overspread this kingdom, in common with most of the countries of Europe and Asia, hospitals dedicated to St. Lazarus, and subsequently bearing his name, were erected for the isolation and maintenance of persons affected therewith; traces and traditions of which have descended to the present day. The Celtic manuscripts contain many references to leprosy and lepers in the middle ages; but these notices chiefly refer to miraculous cures, said to have been performed upon persons affected with that disease, or to the districts and places set apart for their abode.

The present "Leper Hospital" of Waterford, the establishment of which has been attributed by tradition and the local histories to king John,—was originally used for persons labouring under the disease the name of which it bears. There was one leper in it in the year 1775, and that is the last instance of the kind recorded in this country.

Archdale, in his *Monasticon Hibernicum*, gives an account of the following institutions of this nature.

The hospital of St. Stephen, at Waterford, was founded for lepers, and endowed by the Power family. This hospital must have been erected before the Benedictine abbey, for John, Earl

I 2

of Morton, in his charter to that abbey (A.D. 1185), confirmed the leper-house to the poor of the city.

About the end of the twelfth century, according to the commissioners, Alured le Palmer founded an hospital for the sick, in connexion with the priory of St. John the Baptist, in Thomas Street, Dublin; an institution similar in character to the hospital in connexion with the priory of St. Bartholomew, in London, erected by Rahere in 1102. In 1374, the king, "considering the exility of the house and its endowments, and the support of so many poor and sick as were maintained therein, ordained that the prior should, 'during our good pleasure', be exonerated from coming to the marshes with his posse and tenants", etc. "In this house", writes Archdall, "was an infirmary which contained fifty beds for the sick".

During the seventeenth century, the only provision made for the cure of the sick in Ireland, appears to have been intended for the soldiery. One of the historians of the period alludes to "the great hospital of Belfast", in which "there were 3,762 that died in it from November 1st to May 1st, as appears by the telling given in by the men that buried them.

The first modern civil hospital in Dublin was opened in 1726, by six medical men of that city, in Cook Street, and was capable of accommodating four intern patients. It was removed to the Inn's Quay, and is now located in Jervis Street, from which locality it takes its name.

Afterwards the sick hospital took, as it were, a new phase; but for its origin, without reference for the moment to its usefulness or uselessness as an institution, we are undoubtedly indebted to the hierarchy of the Catholic Church. This origin gave weight to the institution, sanctity at one time, and privileges of no mean stamp at all times. The hospital became a kind of church itself, against which, even to these days, the whole army of tax collectors, notwithstanding their increasing encroachments, have not dared to raise a finger.

There is no very clear historical evidence when the Faculty of Medicine was first recognized as of service in hospital practice. In the Palestine hospitals in the chivalrous era, the knights and brothers were the chirurgeons, and in imitation of more ancient heroes, these bound up wounds and invented balsams. The famous *Baume de Commandeur* is a specimen of a cure-all of those days.

The first known hospital to which physicians were attached, belonged to the Templars, under the government of John de

Lastri, who in 1437 undertook the office of grand-master, and defined clearly the duties of physician and surgeon. The application of the sick hospital to the purpose of practical instruction for students, seems to have been established in connexion with a monastery of some Nestorian priests in a town of Persia, called Gaudisapora. Here a medical school was established as early as the seventh century, and became very famous. It is probable, however, that priests only had command of the hospital, and that students were allowed only to visit the sick as learners under the priests; inasmuch as they had to qualify themselves by submitting to the reading of the Psalms of David and the New Testament, and since many of them in later life rose to important ecclesiastical positions. Such is an outline of the origin of the sick hospital, and of its appropriation to the two purposes of treating the sick poor, and educating the Esculapian brotherhood. The project, at the first benevolent, has never lost an iota of the benevolence of its intention, though it may have lost much of the enthusiasm and the romance that marked it in the early periods. In respect to the benevolent intent, the project was excellent. In respect of action, it has been throughout a partial failure and a partial success. A great many lives have been saved by hospitals, a great many sacrificed. The balance is a fine one; we would fain put on the blind of justice while we take it.

As it was in the beginning it is now. Ages have worn away, and generations of the whole earth have died since the sick hospital arose near the homes of men. A grand fundamental mistake of the fathers pertains to the sons. Still, wherever human dwelling places have been most thickly set, the hospital has appeared at hand or in the midst. Nay, the rule has exceeded its origin; for now the sick hospital, limited in its own area, and surrounded by miles of living men, who look towards it as the focus of all their unnecessary and induced physical infirmities, stands bathed in the breath of the uncleansed million, a last mark of an effete barbarism and an age of charitable superstition. We close the windows of this enclosed refuge, and it is forthwith poisoned from within. We open them, and it is forthwith poisoned from without. We place hardy plants within its walls, the plants die. We place sick men in its wards, and we expect them to recover. It is strange, how the minds of men cling to origins. It is not until this age, that true ideas of the true meaning and use of the sick hospital have become known. Even this age is awakening but slowly to knowledge in this regard. The hygiene of hospitals is, as yet, an open subject for discussion, and not altogether a safe subject for those who open the debate. Throwing overboard, for our own parts, all fears of this nature,—all old prejudices, born almost with us and nurtured, certainly, through every day of the student life, —we shall approach this question of hospital hygiene with that freedom which it is our right and duty to exercise in the sphere of our public labours.

It will be gathered from the above, that we consider the present sick hospital system in need of some great and radical reform. It is true; and the points we would urge in the way of this reform are—

1. That intramural hospitals are, in a sanitary point of view, a mistake.

2. That the construction of hospitals, in this country, requires to be immensely simplified and immensely improved.

3. That hospitals for certain specific disorders are, under their existing conditions, charities seriously misapplied.

In relation to the existence of our present intramural hospitals, especially in large towns, the arguments in their favour are two in number. The first is, that the hospitals are standing. The second, that the hospitals are convenient for the sick poor. The first argument is one that need not, to say the most, be perpetuated. The second argument, twenty years ago, when conveyance was difficult and costly, was irresistible. It is feasible even now. Another ten years and it will be a fallacy altogether.

The arguments *against* intramural hospitals are, first, that such hospitals are deprived of all the advantages arising from a constant renewal of fresh and pure air; secondly, that they do not receive the full share of the sunlight; thirdly, that the limited space allotted to them in proportion to their size enforces the necessity of a vicious system of construction; and fourthly, that the deficiency of airing and recreation grounds prevents the application of two of the most effective remedies in the treatment of disease—exercise and pleasurable recreation. The fact that the rural hospital presents a lower mortality than the urban hospital has long been recognized; but in England, as a statistical question, this matter demands a more rigorous investigation than it has received. The *Medical Times and Gazette*, in a late endeavour to encourage the construction of rural hospitals, gives from Dr. Farr the following table:—

FOUR RURAL HOSPITALS.

| Hospitals. | Dates. | One year. In-patients | Mean No. of In-patients | Deaths. | Per Cent. | Dths. in 36 days out of 160 Patnts. |
|---|---|---|---|---|---|---|
| Salop . . . . . | 4 yrs. 1830-33 | 955 | 92 | 34 | 3·7 | 3·6 |
| Winchester | 1 yr. 1833-34 | 798 | 83 | 31 | 3·8 | 3·7 |
| Salisbury . . | 1 yr. 1833-34 | 894 | 88 | 27 | 3·1 | 3·1 |
| Chester . . . | 2 yrs. 1833-35 | 489 | 45 | 20 | 4·2 | 4·5 |
| FOUR CITY HOSPITALS. | | | | | | |
| Manchester | 1 yr. 1831-32 | 1,784 | 128 | 128 | 7·2 | 10·0 |
| Liverpool . . | 1 yr. 1831 | 1,960 | 220 | 109 | 5·6 | 5·0 |
| Bristol . . . . | 1 yr. 1828-29 | 1,483 | 195 | 160 | 9·5 | 8·2 |
| London . . . | | 18,740 | 2,191 | 1,605 | 9·0 | 7·6 |

The table is instructive, but must be received, as our contemporary observes, with proper qualification; for the mortality varies according to the size of an hospital, being greater proportionately in the larger than in the smaller hospitals, and being very much modified by the occurrence or the non-occurrence of accidents or of epidemic visitations in its neighbourhood. On the subject of salubrity, Mr. Roberton speaks with his usual precision. He shows that an unhealthy hospital will unquestionably exhibit a high rate of mortality, but that a high rate of deaths is not always a correct test of the sanitary condition of the building. Thus in a town where people are much employed in machine shops and foundries, the hospital *there* will be sure to receive a larger proportion of severe casualties—of cases likely to swell the death rate—than will happen in another town where few are occupied in connexion with machinery.

The first thing to be regarded, according to this author, in an inquiry into the good or bad sanitary state of any hospital, is, the health of the resident officers and nurses. The second and more decisive test is the tendency in wounds received by accident or by the knife of the surgeon to heal speedily or otherwise—to heal or not to heal as quickly as would happen were the same cases treated singly in a commodious private house.

We agree with Mr. Roberton in his opinion as to the mode of inquiry into the salubrity of hospitals, and maintain that it is by such standards that the relative merits of the rural and town hospital are to be ascertained. And, if this test be admitted, the relative merits of the two classes of hospital can easily be given. The statist's work may, it is true, not appear, but the general experience of the surgical and medical world is too decided in favour of the rural hospital, despite its indifferent construction, to be for a moment gainsaid. Nothing, indeed, is more lamentable than the history of the epidemic outbreaks which now and then visit our large hospitals, and the mortality after surgical operations in hospital practice. The report of the commissioners into the sanitary state of the army brings out important proof of this. In his evidence as to the condition of Guy's Hospital, Dr. Steele states that in the surgical wards pyæmia and erysipelas are common visitants. But Guy's Hospital is by comparison one of the healthiest urban hospitals.

We need not push this point further ; for it is but to question the common sense of mankind to press into notice the facts, that where flowers bloom and all nature rejoices under the light of the lifegiving sun ; that where landscape and skyscape alike rivet the eye and gladden the heart, that there the halting traveller between life and death will find a better haven, than in a dreary corridor with neighbouring misery for his constant companion, with no diversion for his wearied thoughts, with not one moment of fresh breeze to fan his brow, with light half extinguished and landscape and skyscape shut off by miles of brick screen work, and with air for his breath the very presence of which is poison to the hyacinth or the rose.

To make our great hospitals what they ought to be, not a slight on this age but an honour to it, their removal out of the busy city is the first desideratum. But there is yet a kind of perverse idiotcy amongst hospital builders. Instead of looking for a hospital site that shall give to every patient half an acre of ground, your utilitarian charity hospital founder buys half an acre of ground, covers it with brick, and then calculates how many persons he can decently stow into his building, so as to swell his charity to splitting point. He adapts the man to the ground, instead of the ground to the man. His perversity goes further. He looks out in the heart of the great city the most densely populated, most poor and most occluded part, for the small spot which is to be so charitably stocked with sufferers, and advertises daily in the *Times* and elsewhere, to mention nothing about what he says at public dinners, that therefore and because this neighbourhood is so dense and so miserable, so is a hospital required in the centre of it, that misery may concentrate. To make the charity more imposing, he adds massiveness to his scheme. Everything must be massive. The building an enormous mass, strong at base and built very high into the heavens, because into that vault charity extends cheaply in the shape of brick and mortar. His expenses must be immense, £60 per day at the least. His servants must be an army; his advertised admission list of patients per year a veritable county of no mean size. Step forward, my poor friends, step forward into the health mill and through it. Out of it "cured, relieved, unrelieved or dead." This is an institution that refuses none, except when it is chokefull and no more beds can be improvised.

To be a holder of extensive charity shares in a leviathan institution such as ours is to belong to something extensive, says the self-satisfied charity promulgator. Extensive certainly! but extensive in a way that is false to sanitary science, a farce to true philanthropy, a something in which pure charity is least visible, a mere sounding brass and a tinkling cymbal. For what is the object of a hospital which is the refuge and generating point of diseases from which no art or science can rescue the victim? What would be said of a hospital in which the officials are allowed to breed in certain of the wards a race of hydrophobic dogs to lick the wounds of the wounded and keep up a mortal scourge? What would be said? why just what ought to be said to those hospitals where pyæmia and erysipelas are allowed to breed in certain wards and inflict a scourge on the wounded as mortal as mortal mad dog could implant. What should be said? Why, that if these pests are not removable from the ward, then living men, wishing to be cured, should not be moved into such receptacles, and that the grand semblance of charity should be removed at all risks, that the simple reality may be sustained.

Hospitals for large cities to be of unmistakable value to the poor populations, in obedience to sanitary science, and the fundamental laws of the healing art, can only exist in suburban situations. Receiving houses, in connexion with the extramural hospitals, should certainly remain in the town for the temporary reception of accidents; and dispensaries might continue, for giving advice and prescriptions to the sick poor resident at their own dwellings; but the hospital, with its bed-stricken population, has no more business to be crammed into a city than a city has business to be crammed into a hospital.

The first hospital reform, truly hygienic in character, is then, we repeat, removal to an extra urban site. The next question relates to construction. The construction of a hospital has engaged the attention of many thinking men. The views of different men seem, at first sight, to be very conflicting; but, on close analysis, there is really but very little difference in principles. There is now an almost universal acknowledgment that ample space around the hospital is an essential part of any new system. We believe ourselves that the primary consideration in the selection of hospital sites, is to secure such ample grounds about the institution that every hospital having one hundred beds should stand in a plot of favourable ground of at least fifty acres. The soil should be gravelly, and the products of vegetation abundant. The grounds should be laid out artistically, so that the mind of the convalescent man may be constantly in contact with interesting and pleasing objects. The hospital itself, in lieu of being built in one immense edifice, should consist of separate blocks distinct from each other, and each containing at most three wards. The wards themselves should in no way communicate with each other, but be entered by doors communicating indirectly with the open air. The wards should have windows facing each other on all sides, so that currents of air may find their way through and through in all directions. The windows should reach from the bottom to the top of the wards, and should be as numerous as the beds contained in the ward itself. The beds in each ward should not exceed twenty in number, each bed having at least 1,500 cubic feet of air. By a covered passage, each sleeping ward should communicate with an extra room, for daily use, so that the patient might be removed in the day from the room he has occupied during the night. The latrines, as Mr. Roberton observes, should be joined with the wards, so that the sick can pass into them without risk of taking cold, and so placed as to have no ventilating communication with the ward. In St. John's Hospital, Brussels, the latrine is entered from the ward by two doors, an inner and an outer, and the latrine forms a separate building. For purification, the seats are against the outer wall, and the position of the two windows secures a thorough ventilation of the apartment in a direction at right angles with the passage from the ward into the latrine. These arrangements are considered by Mr. Roberton as perfect in their way. The removal of the soil of the latrine is imperative, and for this purpose the self-acting water-closet is much best. It secures permanently a cleansing which would too often be omitted if left to the will or judgment of different persons.

In some hospitals the stench arising from the dressing of wounds is removed, by a contrivance for suddenly flooding the ward with air, and making the current of air to sweep from above downwards and outwards, by openings in the lower part of the wall of the ward. We quote from Mr. Roberton's ventilation the following account of this very useful proceeding :—

"Capped tubes, penetrating the walls a few inches from the floor, are used in Continental hospitals for the occasional ventilation, or, more correctly, for the occasional flooding with air of a particular part of a ward. This contrivance I noticed for the first time in the Hospital de Bairer, Liege. Every surgeon knows, when the dressing is removed from a burn, a wound, an ulcer, or an abscess, that, sometimes, the fœtor is intolerable; and the same will happen in certain states of disease, when the patient is on the bed pan. How it is in such circumstances, by uncovering the mouth of a tube, four inches in diameter, communicating near the floor with the external atmosphere, and the locality is instantly flooded with fresh air, and that, too, at so low a level as not to incommode the patient; when a tube in the opposite wall is at the same moment uncovered, the flood of air is such as allows of both tubes being speedily closed." (p. 10.)

In France, this plan of abstracting the air from the lower part of the ward—*i. e.* from below the level of the patient's bed, instead of above—is becoming exceedingly common. The claims made for this arrangement are thus summed up by Dr. Sutherland, in his note to the army commissioners, on the ventilation and warming of the Parisian hospitals:—

"It is presumed, by arranging the apertures for the exit of foul air near the floor, that emanations, of whatever kind, proceeding from the sick are prevented from becoming diffused through the air of the ward, and that they are directly carried away from the sick bed, that each patient is enveloped in an atmosphere peculiar to himself, and that in cold weather the lower and colder strata of air in the ward are removed, instead of the higher and warmer strata, which would be the case if the ventilating openings were near the ceiling. In proof of the importance of this point, the well known fact of the higher temperature of the upper strata of air in apartments ventilated from the ceiling is adduced. Thus, in one of the minor theatres of Paris so ventilated, the temperature of the air on the level of the floor was found to be 18·36 cent., and 34·52 cent. at the height of 21 feet, showing a difference of 27° Fah.; while in Lariboisière the temperature is the same throughout the whole cubic contents of the ward. The practical result, as regards the sick, of this method of ventilation is stated to be,—

"1. The disappearance of the odour peculiar to sick wards, especially in those containing wounds.

"2. The complete disappearance of epidemic erysipelas, hospital gangrene, and phagedæna, while these diseases still show themselves in wards which have not been ventilated in this manner."

There are three methods for ventilating in this manner, each of which provides for warming also. These are the methods of Duvoir, of MM. Thomas Laurens, and Grouvelle, and of Dr. Heicke. We have described the principle of these plans in previous numbers of this *Review*.

The report of the army commissioners contains records from upwards of sixty hospitals, relative to the different modes of ventilation adopted in hospital practice; and scattered throughout the report are numerous estimates as to the amount of air which is, or ought to be, supplied by certain artificial systems. One inventor boasts that he can give 2,500 cubic feet of air per bed per hour; another, more. Various witnesses also explain that a certain amount of cubic space, 1,500 cubic feet being the most approved figure, are required per bed in the hospital. We agree to this latter proposition, but to the former have only to say that when one man begins to measure out air to another man like a yard of broad cloth, his science is far ahead of his common sense. A ward with windows on each side and at each end, and with a good open fire-grate, requires no measurement of air, nor pump, nor fan, nor steam-jet, nor heated pipe, nor meter trap. The air will blow as it listeth, and ventilate in its way. But it is an advantage to natural ventilation to have a small ward through which the air can circulate freely, and be renewed without cessation.

The mode of artificial lighting of hospitals is a point insisted on by Mr. Roberton, who objects strongly to gas, on account of occasional escape and the glare of the light, and who prefers the oil-lamp. The question of artificial lighting altogether requires remodelling; in hospital, we agree that the oil-lamp is much preferable. One or two moderator lamps in each ward would be found to answer all purposes, and be much less hazardous. The great point, at the same time, to be kept steadily in view in the sick ward is, to do away as much as possible with the artificial light altogether, and to make the patients conform to the eternal law that man shall be the waking and the sleeping companion of the glorious sun.

Regarding the walls, ceilings, furniture and accommodations of hospital wards, the evidence of Miss Nightingale, to which we would especially call the attention of hospital reformers, is as follows:—

"*Qy.* 10,029. What is the best material for the flooring of wards, and your reasons for preferring one material to another?

"The materials used for floors may be oak wood, pine wood, composition, and tiles. Oak wood, well seasoned, is the best. No sawdust, or other organic matter capable of rotting, should be placed underneath the floor. Concrete, or some similar indestructible substance, would be the best for the purpose. The reason for using oak wood is, that it is capable of absorbing but a very small quantity of water. And it is very desirable to diminish even that capability, by saturating it with beeswax and turpentine. Beeswax is an unalterable substance. It is also desirable to avoid the necessity of washing the floor as much as possible. The joints of the flooring must be fitted well together, and cemented with marine glue, or any other impervious substance. The object is, of course, to prevent any water from entering the floor. Impervious, non-absorbent cement, or composition, would make a capital floor, used as it is in Italian houses. But, on account of its great conducting power, it would be necessary to furnish each patient with a pair of list-shoes, and a small bedside carpet. The stairs and landings should be of stone. The corridors should be floored with diamond-shaped flags or tiles, which stand better than those laid in the usual manner. The terrace might be either covered with asphalte or glazed tile.

"10,030. What accommodation for nurses, extra diets, clothing, and clean linen should be attached to the wards?

"There should be a nurse's room, clothes room, a small diet kitchen, and also a store attached to each ward. It is, perhaps, hardly necessary to add that there should be a few small wards for operation cases, and for noisy fever cases.

"10,031. What kind of baths should be attached to the hospital?

"The baths should be separated from the pavilions, but connected by the corridor. The walls and ceilings should be of Parian cement, or some similar material; the floors of tile. They should be suitably ventilated and warmed. They should contain hot and cold water baths, sulphureous water, hot air, medicated and vapour baths and douche. There should be a portable bath to each ward.

"10,032. What is the best form of hospital kitchen?

"The kitchen should be placed away from the wards. Its walls and ceiling should be of Parian cement, for plaster has a tendency to fall off, from the vapour and effluvia of the kitchen. The cooking apparatus, boilers, etc., if placed in the centre of the kitchen, instead of against the walls, will afford twice the amount of fire space. In the Paris kitchens there is a brick erection in the middle of the floor, with iron doors and brass mountings, coppers with covers, places for baking and for roasting, etc. The dressers are against the walls. The floors are flagged with square flags. This appears to be the most convenient mode of erection.

"10,033. What is the best form of laundry for a hospital?

"The excellent new washing, drying, and wringing machines, lately invented, are so numerous that it would take too long to enumerate them. On the whole, the laundry at the Wellington barracks, which also washes for all the Guards' hospitals and barracks, and the new laundry at Haslar Naval Hospital, are the best I have seen. But every day brings in fresh inventions, and a reformer is always adopting the good ones. It is a further question in army matters, whether you should train a body of men to do as much as possible by hand, so as to be serviceable in the field, where machines cannot be had, or whether you should make use of all the inventions for saving labour, which are now so good, and daily improving. Probably both must be done.

"10,034. How should nurses and orderlies be accommodated?

"If orderlies are to sleep among their patients, the percentage of mortality will be, of course, raised among them. This was the case at Scutari, where it was very high, though it will never be known how high. Statistics are, however, not necessary to establish such an obvious fact. The orderlies should sleep at a distance from the wards, and not take their meals in them. With regard to the female nurses, there is the further question as to what class of nurses should be employed in military hospitals. My own opinion is humbly but entirely against employing any but women of the efficiency, responsibility, and character of head-nurses in civil hospitals. For such a nurse a small airy room off her ward, but so that she can always have it under command, is the best for her efficiency, and need not be injurious to her health. Unless, however, there are facile means of access to another nurse's room, in case of illness, there must be only a day-room for each head-nurse adjacent to her ward. She must sleep at a distance from her ward, but contiguous to the other nurses. If assistant female nurses are employed, it must be considered that association in large dormitories tends to corrupt the good and make the bad worse. And separate accommodation, or accommodation for two or three together, should be provided contiguous to the ward, if possible, if not, to one another.

"10,035. What kind of bedstead and bedding is the best?

"No bedding but the hair mattress has yet been discovered that is fit for hospitals. Hair is indestructible. It does not readily retain miasma; and, if it does, heat easily disinfects it. It may be washed. It is not hard to the patient. It saves the objectionable use of a blanket *under* the patient. Straw paillasses are absolutely objectionable. They are cold; and, in some cases, the abstraction of heat from the spine lowers the patient's vital energy to a degree which does not leave him a chance. I am of opinion that the loss of life must have been great during the war from laying our patients on paillasses, which were either placed on wooden divans, or on the flagged corridors, with only a mat between. The bedstead should be iron; and there are great differences in the way the sacking is put in. There should always be a shelf at the head. The French have one at the feet too. The naval and civil hospitals have all kinds of dropsy and surgical bedsteads for raising a patient when he cannot be moved, for inclining him at a certain angle, etc., also water and air beds. There is no reason, but a general objection to comfort, to prevent us using these bedsteads in military hospitals.

"10,036. What ward furniture would you recommend?

"Oak furniture, decidedly. White window curtains are used in some French hospitals, not to exclude the light, but to look cheerful. They are desirable, but not necessary. The less ward furniture, speaking generally, there is the better. But if it be desired by the Commission, I will put it in a list of what is absolutely necessary. Hitherto, as is well known, a military patient has been expected almost to furnish his own ward." (*Commissioners' Report*, p. 382, 383.)

The draining of hospitals and the water supply are equally important questions with those which have gone before. No main drain should pass under the building. Every drain from the building should be kept constantly washed by continuous water-flow and trapped at its outfall. Water derived from a source uncontaminated as far as it can be secured should be at hand in every ward, but procurable only from the original source; or if retained in cisterns, retained in pure cisterns, and of all things again not in cisterns of lead.

As regards construction, the grounds surrounding the model hospital must not be forgotten. These, laid out with taste, should allow of every possible kind of innocent recreation for the convalescent. Gardening, walking, simple games, these are essential. Glass covered walks for wet days are requisites; and miniature tram roads for the easy conveyance into the pure air of the patient unable to leave his bed, these little conveniences are essentials in the true hospital sense, simple though they may appear in themselves. Were proper accommodation made for the conveyance of patients necessarily confined to their beds out of the sleeping ward into the day ward by means of moveable beds, advantages would follow not easily to be described. At the present time, in fact, absolute mischief is done to the sick hospital patient by the circumstance that owing to the necessary limitation of space he is confined almost exclusively to his bed, even though his own instinct and the practitioner's knowledge alike intimate that constant bed is not the treatment. The usefulness of a large hospital would be doubly enhanced as regards admissions if the lingering cases could receive the hygienic treatment of gentle exercise, and if convalescents could secure rapidity of convalescence by the same all potent restorative.

We have neither space nor inclination to dwell at length on the evils of special hospitals as they now exist. Hospitals in which numbers of fever patients or of smallpox patients are all sorted and linked in the bond of residence together. Such hospitals were really too absurd for scientific discourse, were they not concerned in the progress of humanity. They are like the old goals, where the grossest and the mildest of felons were once put together for mutual improvement by more intimate conversation and life. Some of the special diseases of special hospitals are diseases which originally, and as it were naturally, spring up in all localities where air is confined, and the living inmates are numerous and inactive. Enough of this. If correctly built hospitals in rural situations were instituted, special cases would become general, and the treatment which common sense should dictate be secured. Haply then, we might see the typhus-stricken man wheeled out under the open sky, and made to inhale a more certain remedy than ever yet therapeutist gave by mouth, in shape of opiate, salt, or metal.

We have done—yet not for a moment; for the thought will out. In hospital reform lies the whole question of medical scientific reform, which we have so often urged. Why is the arena of medicine such a scene of loud and irregular disputation, broken in upon and in corners ignominiously driven deaf by the voice of quacks and impostors of every grade and kind? Why such a Babel, with each man crying "Eureka", giving to his neighbour but little attention, and expecting not overmuch himself from the moment of his first declaration? Why the clamour?—Because at the great centres of medical education and observation the great facts relating to diseases and their treatment are seen through a haze, and learned loosely in the midst of confusion. Before medicine can stand out immortally and inapproachably, she must give up much, forget greatly what she hath learned, and learn less in a new way. Give up the artificial, pick up the natural, and realize that mere physic is like the operator's knife—the opprobrium of the art.

Thirteen years ago, in the very large hospital of a very large city, there broke out suddenly in the wards, a virulent epidemic of a disease we have already noted—pyæmia. The surgeons put up their knives in fear, and well they might, for to lop off a limb in that building was to put out a life. Out of the hospital, however, in the streets of the city (not a very healthy city, by the way), the surgeons operated as usual and without risk more than common. So the pyæmia had, clearly, a hospital origin, and would not have been in the city but for the hospital, nor in the hospital but for some defect in the hospital itself. The physicians of the hospital, and the surgeons, and the house-surgeons, and the dressers, and the students, and even the nurses, said, What shall we do (not to strike the plague) but to cure the plague-stricken? At the right moment, *i. e.* when confusion was at its highest, one of the house-surgeons, fired by an incomprehensible thought, cried out,

"Try lead." The physicians and the surgeons, catching at shadows, tried lead, therefore, on the spot. The apothecary walking round with the physic basket, dispensed the acetate of lead with liberal hand, and the pyæmics, drawing in long breaths of pyæmic air, took in lead pills by the stomach as the antidote. Whether the lead did good or evil cannot be told; or whether even if a patient had recovered the pyæmia, he would have been affected with after wrist drop, cannot be told, for the poor pyæmics died. But a little later, and a supply of air, improved in quality as in quantity, having been admitted, the pyæmia fled. This event gave rise to the famous local controversy on "Lead *v.* Air, in the Treatment of Pyæmia"; which controversy being scarcely decided as yet, in some quarters, is left with the reader for entire settlement.

---

